# Functional Mapping of Protein Kinase A Reveals Its Importance in Adult *Schistosoma mansoni* Motor Activity

**DOI:** 10.1371/journal.pntd.0001988

**Published:** 2013-01-10

**Authors:** Paulu S. R. de Saram, Margarida Ressurreição, Angela J. Davies, David Rollinson, Aidan M. Emery, Anthony J. Walker

**Affiliations:** 1 Molecular Parasitology Laboratory, School of Life Sciences, Kingston University, Kingston upon Thames, United Kingdom; 2 Wolfson Wellcome Biomedical Laboratories, Zoology Department, The Natural History Museum, London, United Kingdom; Queen's University Belfast, United Kingdom

## Abstract

Cyclic AMP (cAMP)-dependent protein kinase/protein kinase A (PKA) is the major transducer of cAMP signalling in eukaryotic cells. Here, using laser scanning confocal microscopy and ‘smart’ anti-phospho PKA antibodies that exclusively detect activated PKA, we provide a detailed *in situ* analysis of PKA signalling in intact adult *Schistosoma mansoni*, a causative agent of debilitating human intestinal schistosomiasis. In both adult male and female worms, activated PKA was consistently found associated with the tegument, oral and ventral suckers, oesophagus and somatic musculature. In addition, the seminal vesicle and gynaecophoric canal muscles of the male displayed activated PKA whereas in female worms activated PKA localized to the ootype wall, the ovary, and the uterus particularly around eggs during expulsion. Exposure of live worms to the PKA activator forskolin (50 µM) resulted in striking PKA activation in the central and peripheral nervous system including at nerve endings at/near the tegument surface. Such neuronal PKA activation was also observed without forskolin treatment, but only in a single batch of worms. In addition, PKA activation within the central and peripheral nervous systems visibly increased within 15 min of worm-pair separation when compared to that observed in closely coupled worm pairs. Finally, exposure of adult worms to forskolin induced hyperkinesias in a time and dose dependent manner with 100 µM forskolin significantly increasing the frequency of gross worm movements to 5.3 times that of control worms (P≤0.001). Collectively these data are consistent with PKA playing a central part in motor activity and neuronal communication, and possibly interplay between these two systems in *S. mansoni*. This study, the first to localize a protein kinase when exclusively in an activated state in adult *S. mansoni*, provides valuable insight into the intricacies of functional protein kinase signalling in the context of whole schistosome physiology.

## Author Summary

Schistosome blood flukes are formidable parasites. They can survive for many years as male-female pairs in the blood vessels of their vertebrate hosts where they copulate and produce large numbers of eggs that become lodged in tissues causing schistosomiasis. Over 200 million people are infected with schistosomes, mainly in tropical and sub-tropical countries; in terms of parasitic diseases, the socio-economic impact of human schistosomiasis is second only to malaria. Understanding how cellular mechanisms regulate schistosome form and function is a vital part of global research efforts on schistosomes, not least because identification of novel mechanisms might yield opportunities to develop new drugs against the parasite. Here we use a novel approach to provide a comprehensive atlas that displays the localization of an important protein (protein kinase A) when in an exclusively activated state within schistosomes. We show that this protein is activated within various tissues including those of the musculature and nervous system and that its activation in nerves visibly increases when paired adult male and female schistosomes separate. We also show that PKA plays a vital role in the co-coordination of schistosome muscular activity. Our findings offer valuable insight into this protein at the functional level and provide a much-needed physiological framework for further work on PKA in schistosomes, which has been highlighted previously as a potential drug target.

## Introduction

*Schistosoma mansoni* is an important parasitic blood fluke that causes human schistosomiasis, a neglected tropical disease that ranks second only to malaria when considering the number of people infected (∼200 million) and at risk (∼779 million) [1]. The life cycle of this parasite is complex involving snail-intermediate and human-definitive hosts. After the free-living cercariae infect the human host, they transform into parasitic schistosomules which mature in the vasculature *via* an adolescent stage to separate sex adults; sex organs develop approximately three weeks after infection and copulation between male and female worms begins after approximately four weeks [2]. The intimate association that exists between adult male and female worms *in copula* is vital to maintaining the full maturation of the female worm [3]–[5], fertilization of eggs, and thus high levels of egg production to facilitate parasite transmission. Not all of the eggs produced by adult female schistosomes escape from the host. The immune response to those eggs that become trapped in tissues such as the gut wall, liver or spleen and the granulomatous reaction evoked by secretory egg antigens gives rise to chronic/advanced schistosomiasis, with an associated disease burden of ∼70 million disability adjusted life years [6], [7]. Praziquantel is the current drug of choice for the treatment of schistosomiasis but after three decades of use in mono-therapy there remains a possibility that resistance to praziquantel will emerge. Recently the genomes of the three most medically-important schistosomes, *S. mansoni* [8], *Schistosoma japonicum* [9], and *Schistosoma haematobium* [10] were published, providing a valuable resource for integrative biological studies on schistosomes [2] and for identifying potential drug targets [11].

Cyclic AMP (cAMP)-dependent protein kinase/protein kinase A (PKA) is one of the best-characterized members of the protein kinase super-family [12], [13]. In eukaryotes, PKA regulates diverse cellular processes including cell cycle progression [14], proliferation/differentiation [15], [16], cytoskeletal dynamics [17], and flagellar beat [18]. In an inactive state, the PKA holoenzyme comprises two identical catalytic (C) subunits bound non-covalently to two identical regulatory (R) subunits. Activation of PKA occurs in the presence of cAMP that is produced by G-protein coupled receptor (GPCR)-mediated activation of adenylyl cyclase. cAMP binds cooperatively to two sites on each R subunit driving a conformational change within the holoenzyme that results in the release of the catalytically active C subunits enabling them to phosphorylate serine/threonine residues in specific cytosolic and nuclear substrate proteins altering their biological functions [19], [20]. Phosphorylation also plays an important part in the activation of PKA. In mammalian cells, the C subunit is phosphorylated at Thr197 in the activation loop by another C subunit or by phosphoinositide-dependent protein kinase 1 (PDK1) [21]–[23]; in addition Ser338 is phosphorylated, which although not required for enzyme activation is important for processing and maturation of PKA [23]. The broad but selective substrate specificity of PKA is achieved by compartmentalization at different sub-cellular regions through interaction with A-kinase-anchoring proteins (AKAPs) [13]. Furthermore, endogenous protein kinase inhibitor (PKI) peptides inhibit the activity of the C subunit independently of cAMP and also serve to traffic free C subunits from the nucleus to the cytoplasm [24].

In 2009, the first definitive evidence of PKA activity in adult worms was published with a full description of a gene encoding a *S. mansoni* PKA catalytic subunit (Sm-PKA-C) [25]. The putative Sm-PKA-C shared 70% similarity with PKA-C subunits from other organisms including the nematode *Caenorhabditis elegans*, the fruit fly *Drosophila melanogaster*, and *Homo sapiens*, and was most similar to PKA-C of the mollusc *Aplysia californica*. Furthermore, using both RNA interference (RNAi) and pharmacological approaches, PKA expression and activity were found to be essential for schistosome survival [25], highlighting PKA as a possible anti-schistosome chemotherapeutic target.

In the current paper we provide valuable insights into the precise locations and possible functions of phosphorylated (activated) PKA within intact adult *S. mansoni*. Our findings highlight particularly a neuromuscular role for PKA in schistosomes and the detailed analysis of PKA activation within worms provides an important physiological framework for future work on schistosome neurobiology and host-parasite interactions.

## Materials and Methods

### Ethics statement

Laboratory animal use was within a designated facility regulated under the terms of the UK Animals (Scientific Procedures) Act, 1986, complying with all requirements therein; regular independent Home Office inspections occurred. The experiments involving mice in this study were approved by the Natural History Museum Ethical Review Board and work was carried out under Home Office project licence 70/6834.

### Parasites

The Belo Horizonte strain of *S. mansoni* was used in all experiments. Adult schistosomes were recovered by hepatic portal perfusion of female mice (BKW strain) that were infected approximately 45 days earlier by paddling in water containing 200 cercariae. Worm pairs were collected carefully and were either placed immediately in Dulbecco's modified Eagle's medium (DMEM; Invitrogen, Paisley, UK), or were fixed immediately in ice-cold absolute acetone and stored at 4°C for immunohistochemistry.

### Detection of activated PKA in adult *S. mansoni* homogenates by western blotting

Freshly collected adult worm pairs were placed individually in wells of a 12-well tissue culture plate (Nunc, Thermo Fisher Scientific, Loughborough, UK) each containing 1 ml DMEM and were incubated in forskolin (50 µM or 100 µM; Calbiochem, Merck, Nottingham, UK), KT5720 (25 µM or 50 µM; Calbiochem), dimethyl sulphoxide (DMSO) vehicle (0.02% (v/v)), or DMEM alone for 1 h at 38°C. Forskolin was used to activate adenylyl cyclase and produce cAMP to in turn activate PKA; KT5720, a competitive antagonist of the ATP binding site on the PKA catalytic subunit, was employed as a PKA inhibitor. After treatment, each worm pair was homogenized on ice in 25 µl 1× RIPA buffer (20 mM Tris-HCl, 150 mM NaCl, 1 mM EDTA, 1 mM EGTA, 1% (v/v) NP-40) containing 1 µl protease and phosphatase inhibitor cocktail (Pierce; Thermo Fisher Scientific). The resulting homogenate was centrifuged at 13,000 rpm for 10 s at 4°C to remove insoluble material and protein estimations were carried out on the supernatant using the Bradford assay. An appropriate volume of 5× SDS-PAGE sample buffer was added and samples heated to 90°C for 5 min. Once cooled on ice, a further 1 µl of protease inhibitor and phosphatase inhibitor cocktail were added to the extracts and samples stored at −20°C for subsequent electrophoresis. SDS-PAGE was performed using 10% Precise pre-cast gels (Pierce, Thermo Fisher Scientific) and proteins were transferred to nitrocellulose membranes (GE Healthcare, Amersham, UK) using a semi-dry electrotransfer unit (Bio-Rad, Hemel Hempstead, UK). After transfer, membranes were stained with Ponceau S (Sigma, Poole, UK) to confirm homogeneous transfer, and were blocked for 1 h in 5% (w/v) non-fat dried milk in tris-buffered saline containing 0.1% (v/v) Tween-20 (TTBS), and briefly washed in TTBS prior to incubation overnight at 4°C in rabbit anti-phospho-PKA-C (Thr197) polyclonal primary antibodies (Cell Signalling Technology, New England Biolabs, Hitchen, UK; 1∶1000 dilution in 1% (w/v) BSA in TTBS). Next, blots were washed with TTBS and incubated for 2 h at room temperature with horse-radish peroxidase-conjugated secondary antibodies (Cell Signalling Technology; 1∶5000 in 1% BSA (w/v) in TTBS) and exposed to West Pico chemiluminescent substrate (Pierce) for 5 min. Immunoreactive bands were then visualized using a cooled CCD GeneGnome chemiluminescence imaging system (Syngene, Cambridge, UK). Equal loading of proteins was checked by stripping blots for 3 h at room temperature with Restore western blot stripping buffer (Pierce) before briefly washing blots in TTBS and incubating blots with anti-actin antibodies (Sigma, Poole, UK; 1∶3000 in TTBS) followed by secondary antibodies and chemiluminescent imaging. Relative band intensities were quantified using Gene Tools software (Syngene). In addition, to confirm that the anti-phospho-PKA-C (Thr197) primary antibodies only detected the phosphorylated form of PKA-C, western blots were either incubated in primary antibody that had been pre-adsorbed for 30 min to the phosphorylated immunizing peptide or were pre-treated with lambda phosphatase (New England Biolabs; 400 U/ml in TTBS containing 1% BSA and 2 mM MnCl_2_) for 4 h prior to incubation in primary antibodies; secondary antibody labeling and detection were then performed as described above.

### Immunohistochemistry and confocal laser scanning microscopy

Worms processed for confocal laser scanning microscopy included samples fixed immediately after removal from the host and samples fixed after exposure to forskolin (50 µM) as detailed above. Acetone fixed worms were washed twice with 1 ml phosphate buffer saline (PBS) and were further permeabilized with 0.3% (v/v) Triton-X100 in PBS for 1 h. After a brief wash in PBS, worms were blocked for 2 h with 10% (v/v) goat serum (Invitrogen, Paisley, UK) followed by incubation in anti-phospho-PKA-C (Thr197) antibodies (1∶50 in PBS containing 5% (w/v) BSA) for 72 h. Worms were then washed three times in 1 ml PBS for 20 min each and incubated in Alexa Fluor 488 secondary antibodies (Invitrogen; 1∶500 in BSA) and 200 ng/ml rhodamine phalloidin (Sigma) for 24 h in the dark. After further washing with PBS for 1 h, worms were placed on microscope slides and mounted in Vectashield anti-bleaching medium (Vector Laboratories, Peterborough, UK). All washes and incubations were performed in screw-capped microfuge tubes on a microfuge tube rotator at room temperature. Worms were then visualized using a Leica TCS SP2 AOBS confocal laser-scanning microscope using 20× dry objectives or 40x/63x oil immersion objectives and images collected and analyzed with associated Leica software. Because adult *S. mansoni* autofluoresced at the same detection wavelength as the secondary antibody, the signal received from the negative controls (i.e. those not incubated in primary antibody) was negated from the positive samples by reducing the power level of the photomultiplier tube (PMT) and then maintaining constant PMT voltage throughout all observations. Worms were also incubated with anti-phospho-PKA-C (Thr197) antibodies that had been pre-absorbed to the phosphorylated immunizing peptide to check for antibody specificity in immunocytochemistry.

### PKA activation in the nervous system during male-female uncoupling

Freshly collected worm pairs were placed in DMEM at 28°C for 30 min to equilibrate. They were then observed until the first worm pairs uncoupled naturally, and the individual separated male and female worms (five of each) were immediately collected and fixed in ice-cold acetone. At the same times, five coupled worm pairs were removed from the medium and fixed. This provided for analysis worms that were paired and those that had just separated. In addition, immediately upon separation, individual worms were transferred separately to wells of a 24-well culture plate (Nunc) each containing 0.5 ml DMEM maintained at 28°C. These individual male and female worms were then fixed (as described above) at 15 min, 30 min and 60 min post separation (five of each for each time point) to allow for analysis of PKA signalling during pair separation. Similarly, paired worms (five pairs for each time point) were collected, incubated and fixed after these durations to provide paired-worm controls for each separation time point. Fixed worms were then kept at 4°C until they were processed for immunohistochemistry.

### Behavioral effects of PKA activation in *S. mansoni*

Freshly collected adult worm pairs were placed in individual wells of a 12-well tissue culture plate (Nunc) each containing 1 ml of DMEM at 28°C. After 30 min, worms were treated with 50 µM or 100 µM forskolin, or DMSO (0.02% (v/v)) vehicle. Exposing one sample at a time, adult worms were videoed over 30 min using an Olympus SZ4045 binocular dissecting microscope attached to a JVC TK-1481 composite colour video camera operating with Studio Launcher Plus for Windows software with 1 min long movies captured at 0 min, 5 min, 10 min, 15 min, 20 min, 25 min and 30 min post-treatment; videos were compressed using Panasonic dv codecs. A minimum of five worm pairs per treatment were analyzed. During analysis, cold light sources were employed and light intensity kept constant in order to stabilize light condition. The number of gross random muscular movements/min was then assessed visually for each sample at each time point. A gross random muscular movement was defined as a rapid observable change from the existing body position; an extreme example of such movement can be seen with the whip-like motion observed following forskolin treatment in the supplementary movie (Video S1). Next, movies taken for treatments displaying the maximum phenotypic effects were imported into the publicly-available software ImageJ for Windows (Rasband, W.S., ImageJ, U. S. National Institutes of Health, Bethesda, Maryland, USA, http://rsb.info.nih.gov/ij/, 1997–2009) to further assess the nature of worm movement. Prior to import, videos were decompressed to avi format using Movavi Suite 10 SE for Windows and were converted to 8-bit grayscale; background subtraction was performed using ImageJ and the resulting movie was converted to binary format using the automatic Otsu thresholding algorithm. Binary objects representing the worms were then tracked for ‘thrashing’ (body bend) analysis using the open-source publicly-available custom ImageJ plugin, wrMTrck (http://www.phage.dk/plugins) with a bend threshold of 6°. Graphs depicting the angular movement (degrees) of individual worms (shapes) were generated against time (frame number) along with the total number of body bends above the threshold per treatment.

Analysis of variance (ANOVA) and a post-hoc Fisher multiple comparison test were done to analyze the effects of individual treatments at specific time points using Minitab 15.

## Results

### Identification of phosphorylated (activated) PKA in adult *S. mansoni*

**Figure 1:**
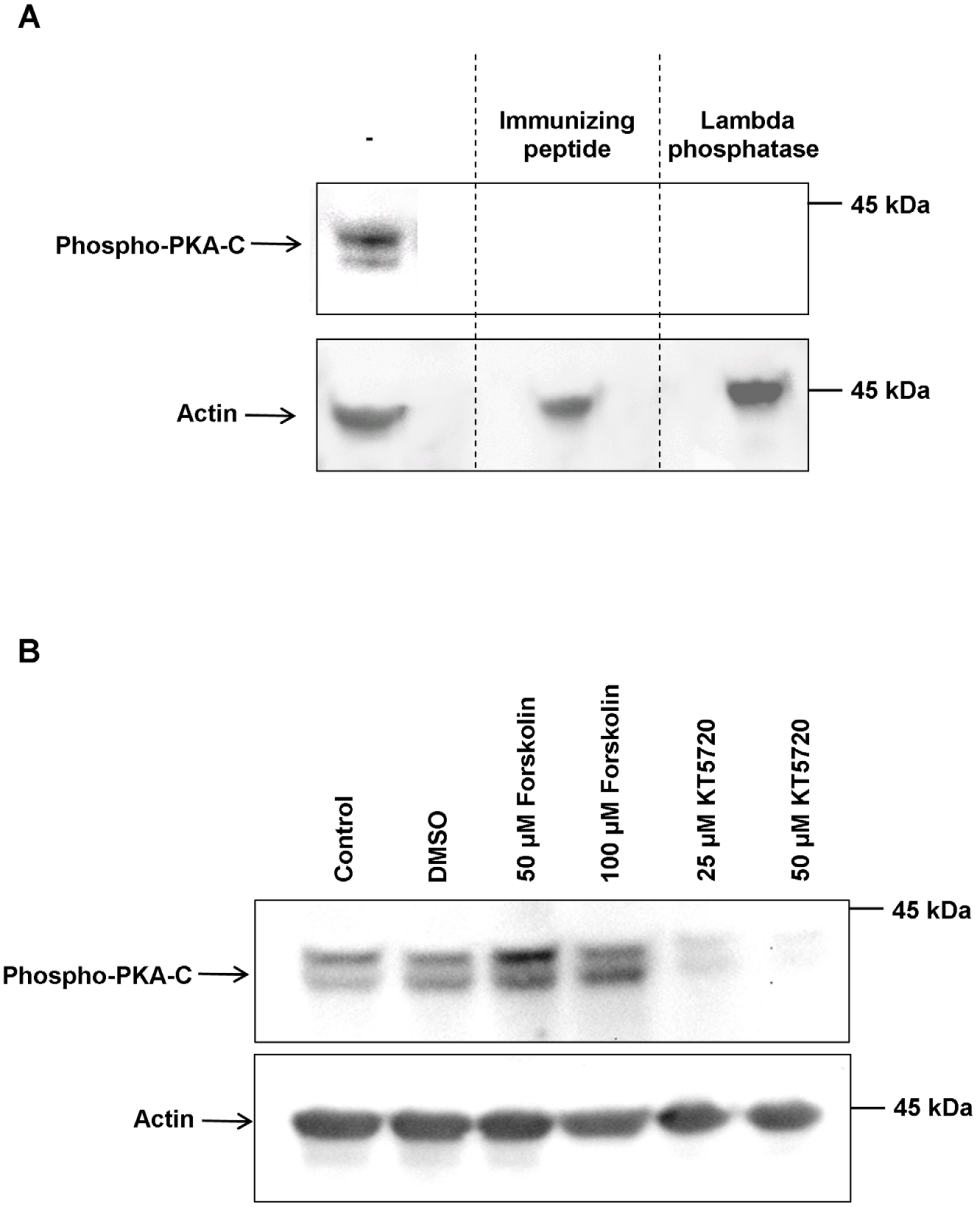
Western blot analysis demonstrating antibody specificity and activation and inhibition of *S. mamsoni* PKA-C. (A) Adult *S. mansoni* protein extracts (from one worm pair) were processed for western blotting with anti-phospho-PKA-C antibodies as described in Materials and Methods; the immunizing peptide and lambda phosphatase were employed to confirm that the antibody reacted with phosphorylated PKA-C and not the non-phosphorylated form. (B) Live adult *S. mansoni* were incubated in 50 µM or 100 µM forskolin, 25 µM or 50 µM KT5720, vehicle (DMSO), or DMEM alone (Control) for 1 h at 28°C and worm protein extracts processed for western blotting with anti-phospho-PKA-C antibodies. Anti-actin antibodies were used to assess any differences in protein loading between samples. The results shown in each panel are representative of data from two independent experiments.

Phospho-specific antibodies that bind to PKA-C only when phosphorylated at a site conserved with threonine 197 (Thr197) of human PKA-C within the PKA activation loop were employed in an attempt to detect phosphorylated PKA in adult *S. mansoni*. Because phosphorylation at this residue is crucial for PKA maturation, optimal conformation and catalytic activity [26], [27], these antibodies are used to determine PKA activation [27]–[29]. Bioinformatic analysis of SmPKA-C [25] comprising 350 amino acids revealed that the amino acid sequence (RVKGRTWTLCGTPEY) surrounding and including Thr197 to which the anti-phospho PKA antibodies bind (www.phosphosite.org) is identical between *S. mansoni* and human PKA, with Thr195 being the crucial threonine phosphorylation site in *S. mansoni* PKA-C. Western blotting revealed that the anti-phospho PKA (Thr197) antibodies detected two closely migrating bands with apparent molecular weights of approximately 40 KDa and 42 KDa in adult *S. mansoni* homogenates (Figure 1A). Treatment of western blots with lambda phosphatase for 4 h prior to exposure to anti-phospho PKA (Thr197) antibodies resulted in a total loss of immunoreactivity of both protein bands; in addition, incubation of these antibodies with the immunizing peptide before exposure to the nitrocellulose resulted in the same effect (Figure 1A). When live adult worms were exposed to the PKA activator forskolin (50 µM or 100 µM) for 1 h the immunoreactivity of both bands increased when compared to controls (Figure 1B). In contrast, exposure to KT5720, a competitive antagonist of the ATP binding site of PKA, decreased the phosphorylation of both bands with 50 µM KT5720 attenuating phosphorylation by approximately 90% (determined by image analysis of two independent bots) (Figure 1B). These results are consistent with the expected effects of antigen competition and dephosphorylation on antibody immunoreactivity and of activation and inhibition on PKA phosphorylation status (e.g. [28], [29]), identifying the anti-phospho-PKA (Thr197) antibodies as suitable for studying PKA activation in *S. mansoni*. The two bands of phosphorylated PKA-C therefore likely represent the 40.4 KDa SmPKA-C characterized by Swierczewski and Davies [25] and an additional PKA-C or splice variant thereof. The *S. mansoni* kinome [30] includes up to five predicted PKA-like proteins, and multiple sequence alignment of these proteins using ClustalW2 (http://www.ebi.ac.uk/Tools/msa/clustalw2/) reveals that the residue corresponding to Thr197 of human PKA-C is conserved in all of these proteins (data not shown).

### *In situ* distribution of activated PKA in adult male and female *S. mansoni*

**Figure 2:**
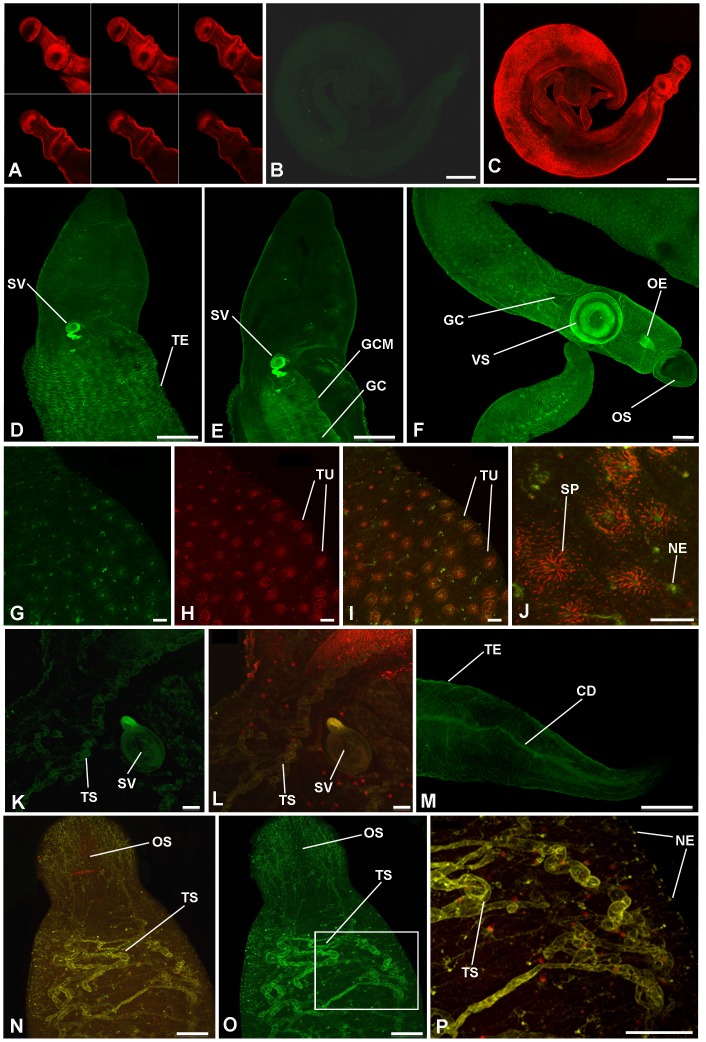
*In situ* distribution of phosphorylated (activated) PKA (green) in adult male *S. mansoni* revealed using anti-phospho-PKA-C antibodies. Schistosomes were fixed and stained as detailed in Materials and Methods. Images show z-axis projections in maximum pixel brightness mode except (A) which shows selected serial optical z-sections; images I, J, L, N, and P are overlays of activated PKA with F-actin stained by rhodamine phalloidin (red). (A–C) Negative control worms incubated without primary antibodies but with Alexa Fluor 488 secondary antibodies and rhodamine phalloidin. In male worms, prominent PKA activation is particularly associated with: (D, anterior dorsal surface scan) the tegument (TE); (E, deeper body scan) gynaecophoric canal muscles (GCM) of the gynaecophoric canal (GC), and seminal vesicle (SV); (F, anterior ventral surface scan) ventral sucker (VS), oral sucker (OS) and oesophagus (OE); (G–J) tubercles (TU) of the tegument, areas proximal to the centres of the tubercle spines (SP) and at surface-proximal nerve endings (NE) are also evident in (P); (K, L, N–P) tubular structures (TS) of unknown function close to the SV (K, L) also in the anterior of the worm (N–P, with boxed region shown in O, enlarged for clarity in P); (M) posterior region displaying activated PKA associated with the collecting duct (CD). Bar: B, C = 300 µm; D–F, M = 100 µm; G–L, P = 20 µm; N,O = 50 µm.

**Figure 3:**
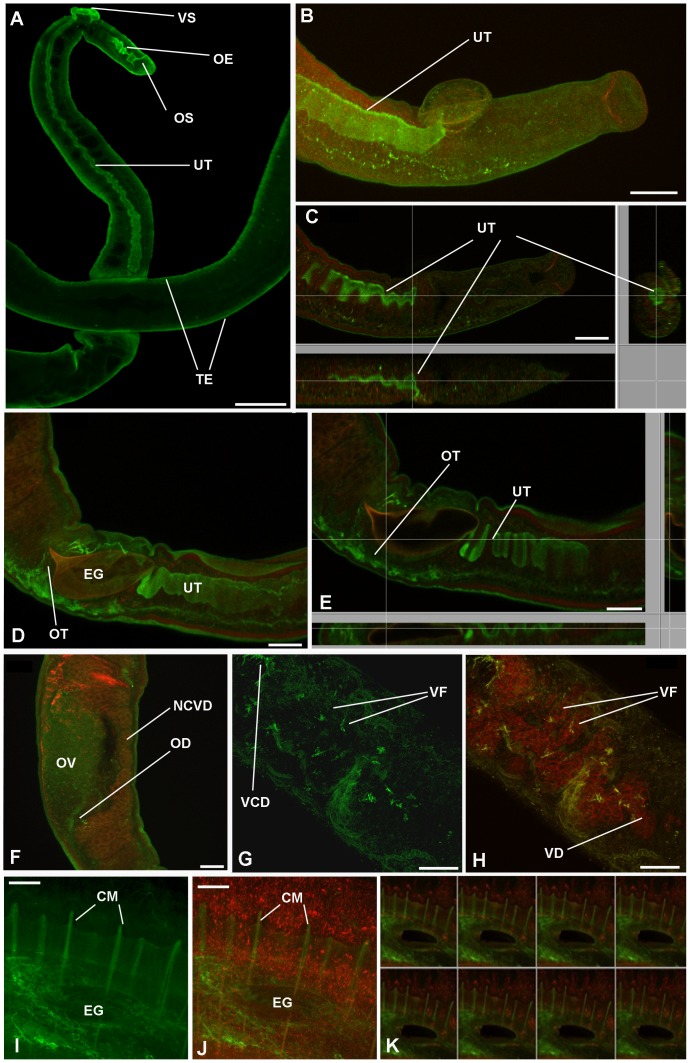
*In situ* distribution of phosphorylated (activated) PKA (green) in adult female *S. mansoni* revealed using anti-phospho-PKA-C antibodies. Schistosomes were fixed and stained as detailed in Materials and Methods. Images show z-axis projections in maximum pixel brightness mode with further z, x and y, z projections in C and E; K shows serial optical z-sections; images B–F, H, J and K are overlays of activated PKA with F-actin stained by rhodamine phalloidin (red). In female worms, prominent PKA activation is particularly associated with: (A) the tegument (TE), oesophagus (OE), ventral and oral suckers (VS/OS); (A–E) uterus (UT); (D, E) ootype (OT) surrounding the egg (EG) which also autofluoresces red in D and E; (F) ovary (OV) and oviduct (OD) but not the non-ciliated vitelline duct (NCVD); (G, H) vitelline collective duct (VCD) structures and areas of the vitelline follicles (VF) leading to the vitelline duct (VD); (I–K, taken from female in copula) circular muscles (CM) revealed as ring-like distributions of activated PKA during egg (EG) expulsion along the uterus. Bar: A = 100 µm; B–J = 40 µm.

To visualize activated PKA in adult *S. mansoni*, anti-phospho-PKA (Thr197) antibodies and confocal laser scanning microscopy were used; all images were obtained from whole mounts of intact worms. Schistosomes that were only incubated in secondary antibody (negative control) displayed almost no fluorescence when autofluorescence was negated by reducing the PMT voltage; only the F-actin staining was evident (Figures 2 A–C). In addition, when adult worms were incubated with anti-phospho-PKA (Thr197) antibodies that had been pre-adsorbed to the phosphorylated immunizing peptide no significant fluorescence was observed, with worms appearing similar to those shown in Fig. 2B. In contrast, incubation with anti-phospho-PKA (Thr197) antibodies revealed activated PKA in various regions of the worms (Figures 2, 3). Intense PKA activation was observed in the tegument of both sexes (Figures 2D, 3A) and this was particularly associated with the tubercles (Figures 2G–2J). PKA activation in the tegument was also more prominent in males than females, and towards the central dorso-lateral region of the male where larger tubercles are present (Figures 2D, 2F; cf. Figure 2M which shows the posterior of the worm). High magnification imaging revealed that regions of the tegument displaying PKA activation included the centres of the tubercles surrounded by spines and foci within the canyons between the tubercles (Figures 2G–J); these regions might represent putative sensory structures in the tegument surface. Furthermore, analysis of serial optical z-sections revealed activated PKA in the musculature immediately underlying the tegument (data not shown). In adult males, deeper scanning revealed activated PKA in the muscular wall of the seminal vesicle (Figures 2E, 2K, 2L), and gynaecophoric canal muscles (Figure 2E) with activation in the latter apparently associated with circular contractile rings (Figure 2E); the oesophagus and the highly muscular ventral sucker also possessed activated PKA as revealed by scanning the ventral side of the worm (Figure 2F). Additionally, activated PKA was visible around an area that resembled the collecting duct of the excretory system at the posterior of the male worm (Figure 2M) and in uncharacterized tubular structures located dorso-laterally between the oral and ventral sucker (Figures 2N–2P); these tubular structures extended approximately to the region where the seminal vesicle is sited (Figures 2K, 2L). In adult female schistosomes and additional to the tegument, activated PKA was associated with the oesophagus, ventral sucker and uterus (Figure 3A), ootype (Figure 3D), vitelline follicles and collecting ducts (Figures 3G, 3H); the common vitelline duct did not possess detectable activated PKA. Deeper scanning and cross-sectional analysis of the uterus and ootype regions revealed activated PKA to be associated with the muscular walls of these organs (Figures 3C, 3E). In addition, in some female worms diffuse PKA activation was detected in the ovary (Figure 3F). Finally, a striking ring-like distribution of activated PKA was detected in the female worm uterus surrounding the egg during egg expulsion along the uterus (Figures 3I–3K); such staining was only observed when an egg was present. Although the rhodamine phalloidin staining appeared diffuse in some cases, analysis of individual channels revealed these ring-like structures to be stained by rhodamine phalloidin thus defining their muscular nature.

### *In situ* distribution of activated PKA in the nervous system of adult male and female *S. mansoni*

**Figure 4:**
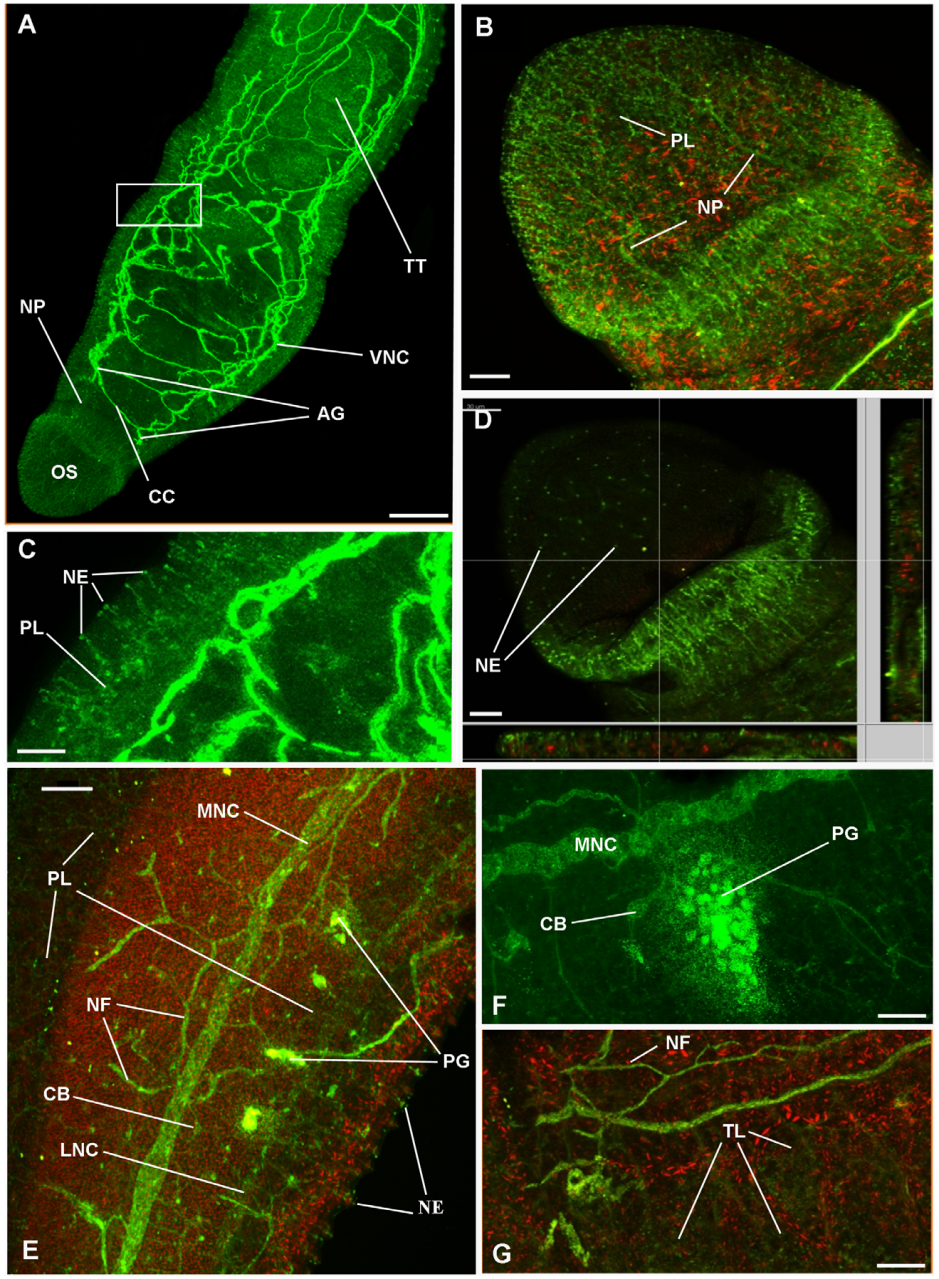
Activated PKA associates with the nervous system of adult male *S. mansoni*. Schistosomes were fixed and stained with anti-phospho-PKA-C antibodies (green) and rhodamine phalloidin (red) to localize activated PKA and actin, respectively, as detailed in Materials and Methods. Images show z-axis projections in maximum pixel brightness mode with further z, x and y, z projections in D. Intense PKA activation is particularly associated with: (A, anterior view) ventral nerve cords (VNC), connecting cerebral commissures (CC), and anterior ganglia (AG); (A, B) complex nerve plexus (PL) associated with the oral sucker (OS) and extending nerve processes (NP); (C, enlarged from boxed region shown in A) and (D) nerve endings (NE) emanating from the PL evident over the body surface including on the tegument and oral sucker; (E, F) peripheral nervous system including PL extending from the lateral nerve cord (LNC), main nerve cord (MNC), complex PL extending to the underlying musculature (red), surface proximal NEs extending from the PL, nerve fibres (NF); peripheral ganglia (PG) and cell bodies (CB) which connect with the MNC; (F) the ganglion shown was close to the surface of the gut. Additionally, diffuse PKA activation is associated with the (A, G) testicular lobes (TL) of the testes (TT), which are surrounded by the testicular capsules stained red. Bar: A = 100 µm; B–D, F, G = 20 µm; E = 40 µm.

**Figure 5:**
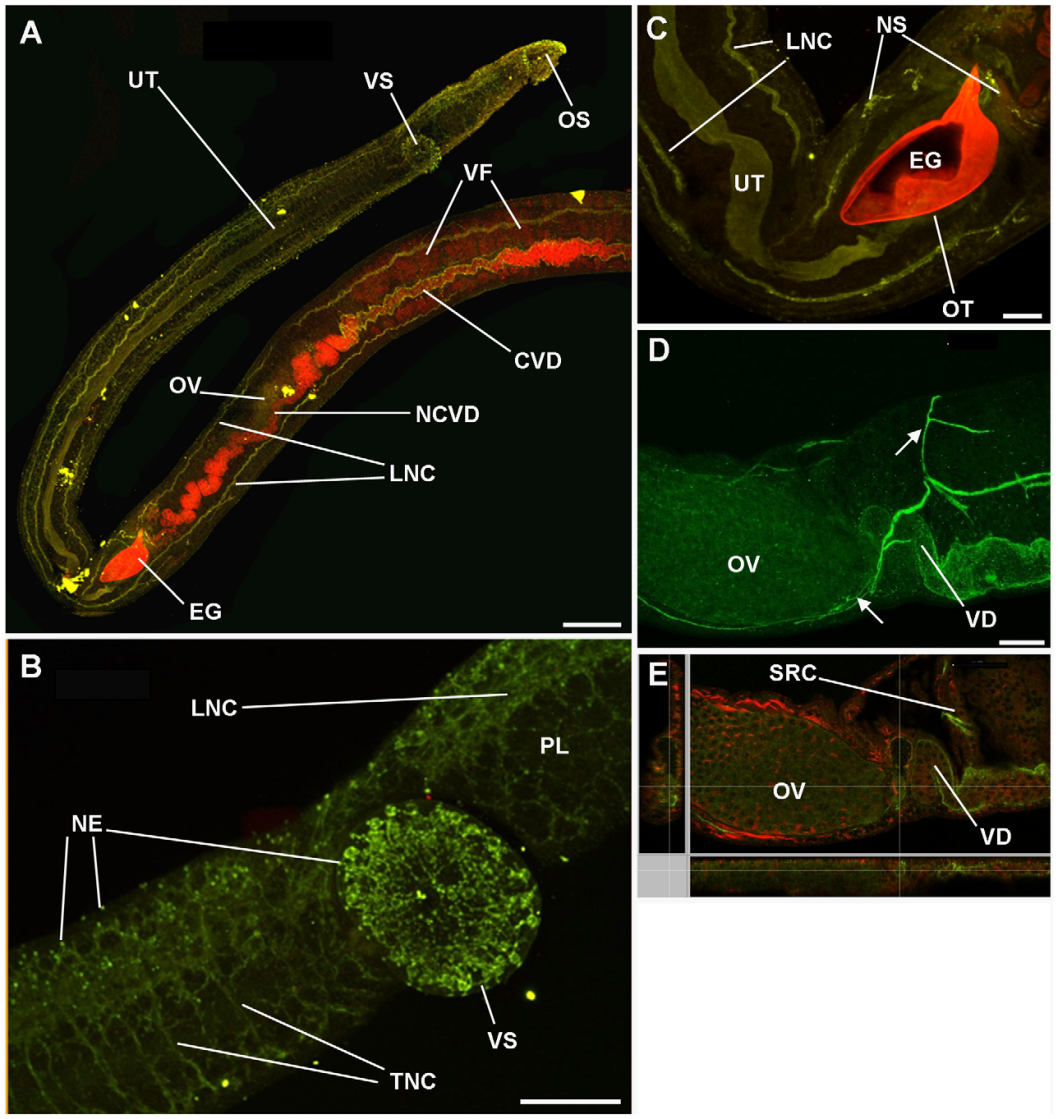
Activated PKA associates with the nervous system of adult female *S. mansoni*. Schistosomes were fixed and stained with anti-phospho-PKA-C antibodies (green) and rhodamine phalloidin (red) to localize activated PKA and actin, respectively, as detailed in Materials and Methods. Images show z-axis projections in maximum pixel brightness mode with further z, x and y, z projections in E. Activated PKA is particularly associated with: (A) longitudinal nerve cord (LNC), neural structures in the oral sucker (OS), ciliated vitelline duct (CVD) adjacent to the vitelline follicles (VF) but not the non-ciliated vitelline duct (NCVD); (B) neural structures of the ventral sucker (VS), surface-proximal nerve endings (NE) extending from the complex nerve plexus (PL) including those on the VS, and transverse nerve cords (TNC); (C) neural structures (NS) innervating the ootype (OT) surrounding the egg (EG), LNC and uterus (UT); (D and E) ovary (OV) and innervating nerves (arrowed) which extend to regions including the vitelline ducts (VD, obscuring the oviducts) and the seminal receptacle complex (SRC). Bar: A = 100 µm; B, D = 40 µm; C = 20 µm.

Normally, activated PKA was not significantly detected in the nervous system of adult male or female worms when paired; however, in about 10% of specimens used (paired and separated) which were all from a single batch of worms collected from a pool of mice from only one infection, striking activation was seen (Figures 4, 5), the general distribution of which was similar between males and females. The basic neuro-anatomy of *S. mansoni* is similar in both sexes; however, the nervous tissue is particularly evident in males due to their larger size. Selective z-scanning to reveal the nervous system demonstrated that activated PKA was present in the anterior ganglia and their connecting commissures and in the dorsal and ventral nerve cords (Figures 4A, 5A); the typical orthogonal arrangement was clearly visible due to activated PKA within the nervous system. Furthermore, activated PKA was detected in the neuropile of the anterior ganglia and connecting commissure that comprised a widespread plexus of nerve fibres (Figure 5B). Activated PKA also existed within the complex innervation of nerve fibres and plexus of the oral sucker and ventral sucker in both sexes (Figures 4A, 4B, 5A, 5B) that originated from the anterior ganglia (determined by analyzing individual z-sections, data not shown). This plexus extended to nerve endings on the sucker tegument surface possessing distinct foci of PKA activation which we propose might have a sensory function (Figures 4B, 4D, 5B); activated PKA was associated with similar nerve endings and underlying plexus over much the worm body (Figures 4A, 4C, 5B).

High-resolution deep body scanning of the male worm peripheral nervous system revealed that activated PKA was also associated with large lateral peripheral ganglia that were positioned between the main nerve cord and more slender lateral nerve cord which both also displayed activated PKA (Figures 4E, 4F); analysis of optical z-sections revealed that some of these ganglia (Figure 4F) were close to the surface of the gut lining (data not shown). Nerve fibres, cell bodies and the complex nerve plexus also stained positive for activated PKA (Figures 4E, 4F). Analysis of z-sections revealed that this nerve plexus served the musculature of the gynaecophoric canal, sub-tegument and tegument surface where activated PKA was seen associated with the nerve endings amongst the spiny tubercles (Figure 4E). Diffuse PKA activation was also seen in the testicular lobes with more evident activation in the nerve fibres around the testes (Figure 4G). In the female, activated PKA was detected in the nerves associated with the ootype and Mehlis' gland complex (Figure 5C) and the ovary and seminal receptacle complex (Figures 5D, 5E).

**Figure 6:**
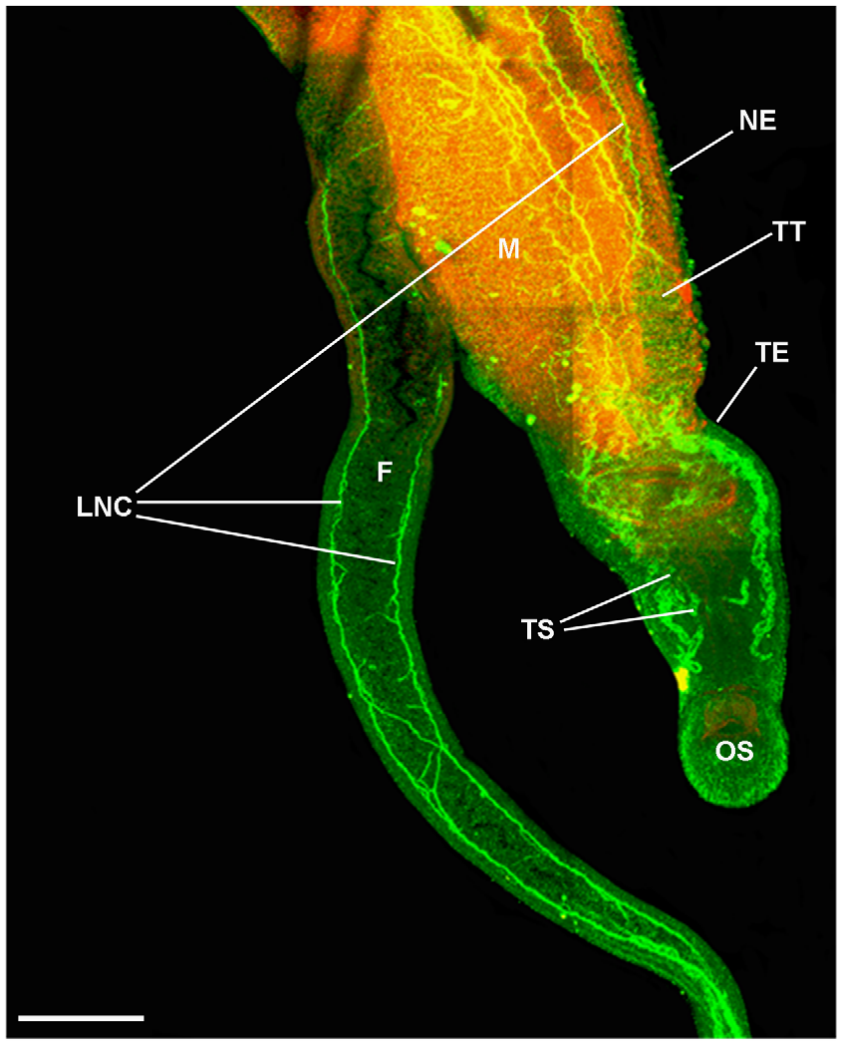
Immunolocalization of activated PKA in forskolin-treated adult male (M) and female (F) *S. mansoni in copula*. Worm couples were incubated in 50 µM forskolin for 1 h and fixed and stained with anti-phospho-PKA-C antibodies (green) and rhodamine phalloidin (red) to localize activated PKA, and actin, respectively, as detailed in Materials and Methods. Intense PKA activation is seen in the tegument (TE) musculature and the nervous system including the longitudinal nerve cords (LNC) and nerve endings (NE) covering the TE and oral sucker (OS). The uncharacterized tubular structures (TS) previously show in Figure 2 also display activated PKA. Image shows a z-axis projection in maximum pixel brightness mode. Bar = 100 µm.

Although extensive PKA activation in the nervous system was only observed in a small proportion of *S. mansoni* recovered, and was not commonly detected, we reasoned that it should be possible to activate neuronal PKA robustly in live *S. mansoni* with 50 µM forskolin. Incubation of worms in forskolin for 1 h resulted in extensive PKA activation in the nervous system (Figure 6), with control worms (not shown) appearing essentially as in Figures 2F and 3A. This increased PKA activation observed within intact worms in response to forskolin mirrors that observed by western blotting (Figure 1B).

### *In situ* distribution of activated PKA during *S. mansoni* uncoupling

**Figure 7:**
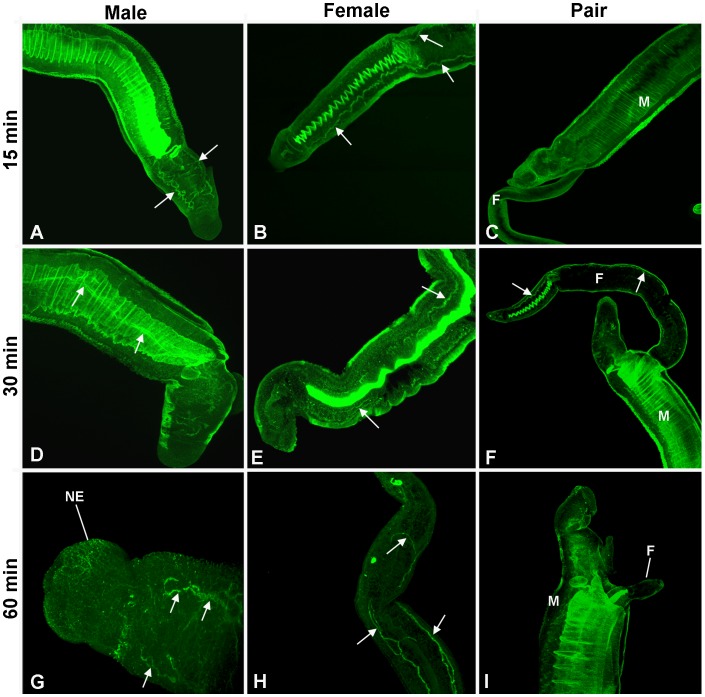
Activation of PKA in the nervous system of adult male (M) and female (F) *S. mansoni* upon pair separation. Schistosomes were fixed (A–C) 15 min, (D–F) 30 min, or (G–I) 60 min after separation and were stained with anti-phospho-PKA-C antibodies (green) to localize activated PKA as detailed in Materials and Methods; schistosomes that remained in copula (pair; C,F,I) for these durations were also processed for comparison with separated males (A,D,G) or females (B,E,H). Images show z-axis projections in maximum pixel brightness mode. The arrows highlight regions of PKA activation within the nervous system; the putative surface-proximal nerve endings (NE) are also indicated.

Although the cause of the extensive activation of neuronal PKA observed in a proportion of worms from only one batch of mice was not known, given the distinct neuronal and muscular distribution of PKA we hypothesized that PKA might become activated in the nervous system during worm un-pairing. In agreement with our previous observations, confocal microscopy revealed that PKA was not activated extensively in the nervous system of paired adult worms immediately after perfusion (data not shown). Increased PKA activation was however observed specifically within the nervous system, including the nerve cords 15 min after pair separation in both male and female worms when compared to their paired counterparts and was sustained for 30 min and 60 min post-separation (Figure 7). Partial PKA activation was also observed in the nerve cord of a female worm that remained *in copula* at 30 min but activation was seen only in areas where the worm had protruded considerably from the male's gynaecophoric canal (Figure 7F); analysis of worms from at least three independent experiments revealed that such neuronal PKA activation was consistently absent when the worms remained closely coupled but was present when they separated. Although it is theoretically possible that separated worms permeabilize more effectively than paired worms, enabling better antibody penetration and labeling, this was not the case in our hands. On occasions when paired worms became separated within rotating tubes during the primary antibody incubation stage no differences in neuronal phospho-PKA labeling could be seen between these paired or separated samples. In addition, PKA activation in the nervous system appeared similar in forskolin treated worms regardless of whether they were paired or separated (data not shown).

### Effect of PKA activation on adult *S. mansoni* movement

**Figure 8:**
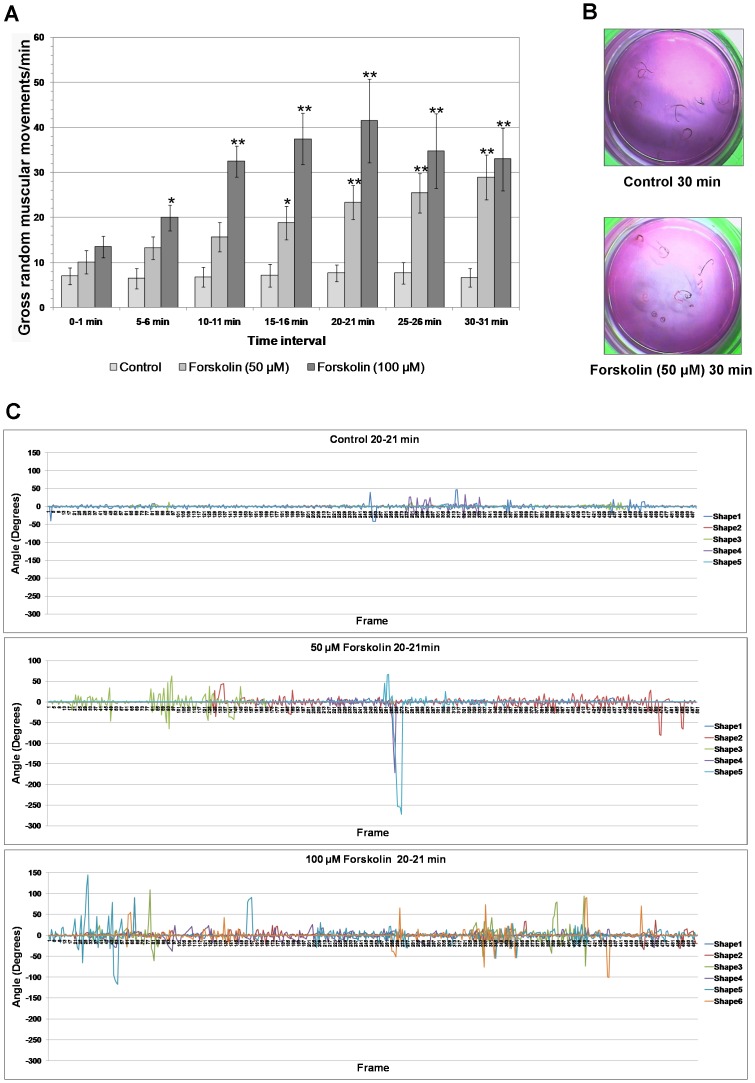
Forskolin increases the frequency and angle of *S. mansoni* adult worm movements. (A) Adult worms were incubated in either 50 µM or 100 µM forskolin, or DMSO vehicle (control) for various durations (0–30 min) and were filmed for 1 min and analyzed as detailed in Materials and Methods. Values shown represent mean number of gross random muscular movements per minute (±SEM, n = 7 for each treatment at each time point); *P≤0.05; **P≤0.001. (B) An increased propensity of worm coiling was observed following incubation with 50 µM forskolin when compared to controls; effects after 30 min are illustrated. (C) Detailed analysis of individual worm (shape) movements using the ImageJ plugin wrMTrck when worms were incubated in either 50 µM or 100 µM forskolin, or DMSO vehicle (control) for 20 min. The angle (degrees) of worm movement is shown as a function of time (frame) during 1 min recording; results of at least 5 individual worms are shown.

Because activated PKA localized to the musculature and nervous system of adult *S. mansoni*, an experiment was conducted to ascertain the effect of PKA activation on worm movement. Preliminary assays revealed that forskolin treatment induced a phenotype that displayed considerable random contractile movements. Movies of adult worms were therefore captured for visual semi-quantitative analysis. When worms were treated with 50 µM forskolin there was a significant increase in gross muscular movements with time (*P*≤0.001; Figure 8A). After 15 min, the mean number of gross muscular movements in control worms was 7.2/min whereas the frequency in forskolin treated worms increased 2.6 times to 18.9/min (*P*≤0.05). This forskolin-mediated effect was even more pronounced after 20 min (*P*≤0.001) and was sustained until the end of the experiment (Figure 8A) by which time movements had increased to 4.3 times that of control (*P*≤0.001). Forskolin-treated worms also displayed excessive ‘coiling’ when compared to their non-treated counterparts and this was particularly evident after 20, 25 and 30 min exposure (30 min shown in Figure 8B). The effects of 100 µM forskolin on gross random muscular movements were more pronounced than with 50 µM forskolin, showing a significant increase after only 5 min (*P*≤0.05) when compared to controls (Figure 8A). After 10, 15, and 20 min, the frequency of movements was significantly greater in the presence of 100 µM forskolin than in 50 µM forskolin (*P*≤0.01), and after 20 min exposure worms displayed 41.5 movements/min compared to only 7.7 in the control group (Figure 8A; *P*≤0.001). Despite the increased motility observed in the presence of forskolin, there was no apparent difference in the number of worm pairs that separated during the course of the experiment. The movie (Video S1) provides a visual inspection of the effects of forskolin treatment on worm movement at 20 min exposure. Movies of worms exposed to forskolin for 20 min were then subjected to quantitative thrashing (body bend) analysis using the ImageJ plugin wrMTrck. Both the number of body bends and the extent of angular movement increased considerably following exposure to either 50 µM or 100 µM forskolin when compared to DMSO controls (Figure 8C), with irregular movements observed. Using a threshold of 6° change, the average number of body bends per worm following exposure to these concentrations of forskolin was 4.6 and 6.9 times that of controls, respectively, and was broadly similar to that determined for this time point by semi-quantitative analysis (Figure 8A). Importantly, wrMTrck analysis also revealed the extent of change in angular movement of worms following treatment. Whereas thrashing in excess of ±25° was infrequent in controls, it was considerably more common with forskolin (Figure 8C). Moreover, thrashing in excess of ±50° did not occur in controls, but did on eight occasions with 50 µM forskolin and 24 occasions with 100 µM forskolin. Collectively, this data highlights the nature of the hyperkinetic effect of forskolin and thus PKA activation on *S. mansoni* worm movement.

## Discussion

By using anti-phospho-specific antibodies and laser-scanning confocal microscopy we have mapped in detail activated PKA, the major transducer of cAMP signalling in eukaryotes [31], to discrete tissues of intact male and female adult *S. mansoni*. In male worms, activated PKA was found particularly associated with the tegument, gynaecophoric canal muscles, oral and ventral suckers, oesophagus, seminal vesicle wall, other areas of somatic musculature, and anterior tubular structures of unknown function. In females, activated PKA was observed particularly in the tegument, suckers, oesophagus, uterus and ootype wall, and ovary. In addition, in a subset of worms obtained from a pool of mice all from the same infection and in forskolin-treated worms, striking PKA activation was observed throughout much of the central and peripheral nervous systems including at nerve endings at the worm surface. Activation of neuronal PKA appeared to increase during worm separation *in vitro*, and pharmacological activation of PKA by forskolin induced hyperkinesias in worms in a time and dose-responsive manner. Collectively, these data are consistent with PKA likely playing a vital part in *S. mansoni* muscular activity and neuronal communication, and interplay between these two systems.

Schistosomes employ a variety of biogenic amines (e.g. 5-hydroxytryptamine (5-HT/serotonin), dopamine and histamine) and neuropeptides in their nervous system [32], [33]. Biogenic amines signal through GPCRs and in some cases activate adenylyl cyclase, elevating intracellular cAMP levels which in turn activate PKA [34], [35]. 5-HT increases the motility of intact schistosomes *in vitro* [e.g. 36]–[38] and has been localized to the male gynaecophoric canal and oral and ventral suckers [37] with a distribution similar to that seen with activated PKA in the current study. Other GPCRs such as SmGPR-3 [39] and SmD2 [40] which are activated by dopamine and are expressed in the central/peripheral nervous systems and body wall musculature, respectively, might influence *S. mansoni* movement in a complex fashion given that dopamine suppresses *S. mansoni* motor activity [39], [41] but induces cAMP production *via* SmD2 [40]. Furthermore, l-glutamate induces muscle contraction in isolated *S. mansoni* muscle fibres [42] likely via l-glutamate receptors [43] and kainic acid, an agonist that mimics the effect of glutamate, causes hyperkinesias and coiling in adult worms [44] similar to that observed with forskolin in the present study. Motor activity in *S. mansoni* thus appears to be under complex regulatory control from neuromodulators and classical neurotransmitters some of which will likely signal to PKA. Our findings should thus help drive forward research aimed at elucidating some of the crucial downstream signalling mechanisms that govern muscular activity which is central to parasite survival and reproduction in the host. It is important to note, however, that the mechanisms by which PKA influences muscle contraction and relaxation in mammals are complex and are not fully understood (see for example, [45]). Elucidating mechanistic control of motility in schistosomes will therefore require significant endeavor.

Extensive PKA activation was evident in the muscular walls of the uterus and ootype. The ootype which comprises regularly arranged circular and longitudinal muscle fibres [46] is the site of egg formation where an egg is produced from a fertilized ovum, with secretions from the vitelline cells and Mehlis' gland. The uterus, which possesses mainly circular fibers [46] leads from the ootype to the genital pore and opens close to the ventral sucker of the female. Swierczewski and Davies [25], [47] demonstrated that the PKA inhibitor H-89, or PKI 14–22 amide, significantly impaired egg output by *S. mansoni*, and when used at 10 µM or higher, H-89 stopped egg production during the first day of observation. Our findings suggest that active PKA helps regulate *S. mansoni* muscular activity and thus coordinated activation of PKA in the ootype wall could be vital for successful egg formation. In addition, because activated PKA was observed around eggs within the uterus during egg expulsion and also in ring like structures that circumscribed the egg, PKA activity could be necessary for egg propulsion, a process presumably enabled through peristaltic movement. Thus, the effects of PKA inhibitors on egg output by female worms observed by Swierczewski and Davies [25], [47] may have been, at least in part, due to blockade of egg formation in the ootype or dysregulated peristaltic movement along the uterus. Swierczewski and Davies [25] also reported that H-89 caused dissociation of worm pairs, although the duration required for separation was not reported. Here, in the presence of the PKA activator forskolin for 30 min, there was no difference in the number of worm pairs that separated when compared to controls despite the hyperkinetic effects of forskolin on worm motility.

Striking PKA activation was observed in an extensive network of nerve endings at the surface of the tegument, including those associated with the oral and ventral suckers. These endings were linked to the underlying nerve plexus associated with the peripheral and central nervous systems, which in some worms also displayed extensive PKA activation. The presence of such an evolutionarily-conserved signalling pathway [31] at tegumental nerve endings is intriguing as it suggests that *S. mansoni* might use these structures to transduce signals from the host and perhaps from other individual worms *via* PKA signalling. In this context, it is interesting that after worm pair separation *in vitro*, PKA activation visibly increased specifically in the nervous tissue, including in the central nervous system; in closely coupled worms, active PKA in the nervous system was essentially absent. From the behavioral stand point this is an important finding as it suggests that worm pairing and maintenance of the *in copula* state may somewhat be governed by sensory neuronal mechanisms mediated by PKA. As recently highlighted [2], integration of cell signalling into research on schistosome sensory biology will help drive forward this important area of research.

The presence of activated PKA in the muscular uterus, oesophagus, suckers, and ring-like structures in the gynaecophoric canal and in the uterus during egg propulsion, the nerves innervating the musculature, and the effects of forskolin on gross worm movements (hyperkinesia) shown here, coupled with un-pairing in the presence of H-89 reported previously [25] support a role for activated PKA in the regulation of *S. mansoni* motor activity. It is worthy to note that PKA possesses wide ranging regulatory roles in animals. For example, in neurons PKA has also been implicated in processes such as protein degradation, protein trafficking and gene transcription [48]–[50]. Indeed, PKA signalling through the nervous system might well be one mechanism by which schistosomes transmit signals to maintain homeostasis. Certainly, the lethal effect of PKA-C knockdown by either RNAi or PKA inhibition [25] signifies the central importance of this enzyme to worm function.

In *S. mansoni* PKA has also been implicated in regulating the ciliary motion of miracidia [51] likely through conserved axonemal mechanisms (discussed by Ressurreicao et al. [52]). In addition, PKA inhibition by H-89 has been shown to speed up the rate of transformation of miracidia to mother sporocysts [53], possibly through the effect of attenuated locomotion and thus early loss of epidermal ciliated plates. Expression levels of PKA-C were recently found to differ between different life stages of *S. mansoni*, and inhibition of PKA by H-89 or PKI 14–22 amide was found to be lethal to cercariae [47] as in adult worms. Our overall knowledge of the signalling mechanisms that regulate schistosome development and function is poor (discussed in [2]) and it is therefore important for future studies to encompass the importance of protein kinases, such as PKA, PKC [54] and MAPKs [55] to the development of definitive host stages particularly in the context of organism function and possible host-parasite interplay. In the case of PKA, consideration should be given to the importance of the regulatory subunits (also highlighted in [25]) and also AKAPs which permit targeting of PKA to certain sub-cellular locations. The current work, which includes a vital atlas of exclusively activated PKA in adult male and female *S. mansoni*, should prove invaluable for future studies into PKA function during worm development, pairing, and host-parasite interactions, as well as for studies into upstream effector mechanisms and downstream target molecules.

## Supporting Information

Video S1 **Forskolin increases the frequency of gross muscular movements in adult** ***S. mansoni*** **.** Adult worms were incubated in either 50 µM or 100 µM forskolin, or DMSO vehicle (control) for 20 min and were filmed for 1 min. In addition to the increased worm movement observed in the presence of forskolin, note the increased tendency of worms to ‘coil’ particularly at the higher dose (100 µM).
(WMV)

## References

[1] Steinmann P, Keiser J, Bos R, Tanner M, Utzinger J (2006) Schistosomiasis and water resources development: systematic review, meta-analysis, and estimates of people at risk. Lancet Infect Dis 6: 411–425. 16790382 10.1016/S1473-3099(06)70521-7

[2] Walker AJ (2011) Insights into the functional biology of schistosomes. Parasite Vectors 4: 203. 10.1186/1756-3305-4-203 PMC3206467 22013990

[3] Popiel I, Basch PF (1984) Reproductive development of female *Schistosoma mansoni* (Digenia: *Schistosomatidae*) following bisexual pairing of worms and worm segments. J Exp Zool 232: 141–150. 6502090 10.1002/jez.1402320117

[4] Kuntz W (2001) Schistosome male-female interaction: induction of germ-cell differentiation. Trends Parasitol 17: 227–231. 11323306 10.1016/s1471-4922(01)01893-1

[5] Gelanti SE, Huang SCC, Pearce EJ (2012) Cell death and reproductive regression in female *Schistosoma mansoni* . PLoS Negl Trop Dis 6: e1509. 22363825 10.1371/journal.pntd.0001509 PMC3283563

[6] King CH, Dickman K, Tisch DJ (2005) Reassessment of the cost of chronic helminthic infection: a meta-analysis of disability-related outcomes in endemic schistosomiasis. Lancet 365: 561–569. 15866310 10.1016/S0140-6736(05)66457-4

[7] Grey DJ, Ross AG, Li YS, McManus DP (2011) Diagnosis and management of schistosomiasis. Brit Med J 342: d2651. 21586478 10.1136/bmj.d2651 PMC3230106

[8] Berriman M, Haas BJ, LoVerde PT, Wilson RA, Dillon GP, et al (2009) The genome of the blood fluke *Schistosoma mansoni* . Nature 460: 352–358. 19606141 10.1038/nature08160 PMC2756445

[9] The *Schistosoma japonicum* Genome Sequencing and Functional Analysis Consortium (2009) The *Schistosoma japonicum* genome reveals features of host-parasite interplay. Nature 460: 345–352. 19606140 10.1038/nature08140 PMC3747554

[10] Young ND, Jex AR, Li B, Liu S, Yang L, et al (2012) Whole-genome sequence of *Schistosoma haematobium* . Nature Genet 44: 221–225. 22246508 10.1038/ng.1065

[11] Crowther GJ, Shanmugam D, Carmona SJ, Doyle MA, Hertz-Fowler C, et al (2010) Identification of attractive drug targets in neglected-disease pathogens using an *in silico* approach. PLoS Negl Trop Dis 4: e804. 20808766 10.1371/journal.pntd.0000804 PMC2927427

[12] Taskén K, Aandahl EM (2004) Localized effects of cAMP mediated by distinct routes of protein kinase A. Physiol Rev 84: 137–167. 14715913 10.1152/physrev.00021.2003

[13] Pidoux G, Taskén K (2010) Specificity and spatial dynamics of protein kinase A signaling organized by A-kinase-anchoring proteins. J Mol Endocrinol 44: 271–284. 20150326 10.1677/JME-10-0010

[14] Zambon AC, Zhang LZ, Minovitsky S, Kanter JR, Prabhakar S, et al (2005) Gene expression patterns define key transcriptional events in cell-cycle regulation by cAMP and protein kinase A. Proc Natl Acad Sci USA 102: 8561–8566. 15939874 10.1073/pnas.0503363102 PMC1150853

[15] Cass LA, Summers SA, Prendergast GV, Backer JM, Birnbaum MJ, et al (1999) Protein kinase A-dependent and -independent signaling pathways contribute to cyclic AMP-stimulated proliferation. Mol Cell Biol 19: 5882–5891. 10454535 10.1128/mcb.19.9.5882 PMC84437

[16] Pan XW, Heitman J (2002) Protein kinase A operates a molecular switch that governs yeast pseudohyphal differentiation. Mol Cell Biol 22: 3981–3993. 12024012 10.1128/MCB.22.12.3981-3993.2002 PMC133872

[17] Liu F, Verin AD, Borbiev T, Garcia JGN (2001) Role of cAMP-dependent protein kinase A activity in endothelial cell cytoskeleton rearrangement. Am J Physiol – Lung Cell Mol Physiol 280: L1309–L1317. 11350812 10.1152/ajplung.2001.280.6.L1309

[18] Nolan MA, Babcock DF, Wennemuth G, Brown W, Burton KA, et al (2004) Sperm-specific protein kinase A catalytic subunit C alpha(2) orchestrates cAMP signaling for male fertility. Proc Natl Acad Sci USA 101: 13483–13488. 15340140 10.1073/pnas.0405580101 PMC518783

[19] Taylor SS (1989) cAMP-dependent protein kinase – model for an enzyme family. J Biol Chem 264: 8443–8446. 2656679

[20] Kim C, Cheng CY, Saldanha SA, Taylor SS (2007) PKA-I holoenzyme structure reveals a mechanism for cAMP-dependent activation. Cell 130: 1032–1043. 17889648 10.1016/j.cell.2007.07.018

[21] Cauthron RD, Carter KB, Liauw S, Steinberg RA (1998) Physiological phosphorylation of protein kinase A at Thr-197 is by a protein kinase A kinase. Mol Cell Biol 18: 1416–1423. 9488457 10.1128/mcb.18.3.1416 PMC108855

[22] Nirula A, Ho M, Phee H, Roose J, Weiss A (2006) Phosphoinositide-dependent kinase 1 targets protein kinase A in a pathway that regulates interleukin 4. J Exp Med 203: 1733–1744. 16785309 10.1084/jem.20051715 PMC2118337

[23] Keshwani MM, Klammt C, von Daake S, Ma Y, Kornev AP, et al (2012) Cotranslational *cis*-phosphorylation of the CooH-terminal tail is a key priming step in the maturation of cAMP-dependent protein kinase. Proc Natl Acad Sci USA 109: E1221–1229. 22493239 10.1073/pnas.1202741109 PMC3356610

[24] Dalton GD, Dewey Wl (2006) Protein kinase inhibitor peptide (PKI): A family of endogenous neuropeptides that modulate neuronal cAMP-dependent protein kinase function. Neuropeptides 40: 23–34. 16442618 10.1016/j.npep.2005.10.002

[25] Swierczewski BE, Davies SJ (2009) A schistosome cAMP-dependent protein kinase catalytic subunit is essential for parasite viability. PLoS Negl Trop Dis 3: e505. 19707280 10.1371/journal.pntd.0000505 PMC2724707

[26] Yonemoto W, McGlone ML, Grant B, Taylor SS (1997) Autophosphorylation of the catalytic subunit of cAMP-dependent protein kinase in *Escherichia coli* . Prot Eng 10: 915–925. 10.1093/protein/10.8.915 9415441

[27] Cheng X, Ma Y, Moore M, Hemmings BA, Taylor SS (1998) Phosphorylation and activation of cAMP-dependent protein kinase by phosphoinositide-dependent protein kinase. Proc Natl Acad Sci USA 95: 9849–9854. 9707564 10.1073/pnas.95.17.9849 PMC21425

[28] Brackenbury WJ, Djamgoz MBA (2006) Activity-dependent regulation of voltage-gated Na^+^ channel expression in Mat-LyLu rat prostate cancer cell line. J Physiol 573: 343–356. 16543264 10.1113/jphysiol.2006.106906 PMC1779734

[29] Chioni AM, Shao D, Grose R, Djamgoz MBA (2010) Protein kinase A and regulation of neonatal NaV1.5 expression in human breast cancer cells: activity-dependent positive feedback and cellular migration. Int J Biochem Cell Biol 42: 346–358. 19948241 10.1016/j.biocel.2009.11.021

[30] Andrade LF, Nahum LA, Avelar LGA, Silva LL, Zerlotini A, et al (2011) Eukaryotic protein kinases (ePKs) of the helminth parasite *Schistosoma mansoni* . BMC Genomics 12: 215. 21548963 10.1186/1471-2164-12-215 PMC3117856

[31] Das R, Esposito V, Abu-Abed M, Anand GS, Taylor SS, et al (2007) cAMP activation of PKA defines an ancient signalling mechanism. Proc Natl Acad Sci USA 104: 93–98. 17182741 10.1073/pnas.0609033103 PMC1765484

[32] Ribeiro P, El-Shehabi F, Patocka N (2005) Classical transmitters and their receptors in flatworms. Parasitology 131: S19–S40. 16569290 10.1017/S0031182005008565

[33] Ribeiro P, Geary T (2010) Neuronal signaling in schistosomes: current status and prospects for post genomics. Can J Zool 88: 1–22.

[34] Noda M, Higashida H, Aoki S, Wada K (2004) Multiple signal transduction pathways mediated by 5-HT receptors. Mol Neurobiol 29: 31–39. 15034221 10.1385/MN:29:1:31

[35] Missale C, Nash S, Robinson S, Jaber M, Caron M (1998) Dopamine receptors: from structure to function. Physiol Rev 78: 189–225. 9457173 10.1152/physrev.1998.78.1.189

[36] Mellin TN, Busch RD, Wang CC, Kath G (1983) Neuropharmacology of the parasitic trematode, *Schistosoma mansoni* . Am J Trop Med Hyg 32: 83–93. 6130710 10.4269/ajtmh.1983.32.83

[37] Withyachumnarnkul B, Pongsa-Asawapaiboon A, Sobhon P, Upatham ES (1987) The neural commissural rings of the adult male *Schistosoma mansoni* . J Helminthol 61: 169–172. 3611711 10.1017/s0022149x00009949

[38] Boyle JP, Yoshino TP (2005) Serotonin-induced muscular activity in *Schistosoma mansoni* larval stages: importance of 5-HT transport and role in daughter sporocyst production. J Parasitol 91: 542–550. 16108544 10.1645/GE-432R

[39] El Shahabi F, Taman A, Moali L, El-Sakkary N, Ribeiro P (2012) A novel G protein-coupled receptor of *Schistosoma mansoni* (SmGPR-3) is activated by dopamine and is widely expressed in the nervous system. PLoS Negl Trop Dis 6: e1523. 22389736 10.1371/journal.pntd.0001523 PMC3289605

[40] Taman A, Ribeiro P (2009) Investigation of a dopamine receptor in *Schistosoma mansoni*: Functional studies and immunolocalization. Mol Biochem Parasitol 168: 24–33. 19545592 10.1016/j.molbiopara.2009.06.003

[41] Pax RA, Siefker C, Bennett JL (1984) *Schistosoma mansoni*: differences in acetylcholine, dopamine, and serotonin control of circular and longitudinal parasite muscles. Exp Parasitol 58: 314–324. 6500002 10.1016/0014-4894(84)90048-1

[42] Miller CL, Day TA, Bennett JL, Pax RA (1996) *Schistosoma mansoni*: L-glutamate-induced contraction in isolated muscle fibres; evidence for a glutamate transporter. Exp Parasitol 84: 410–419. 8948330 10.1006/expr.1996.0129

[43] Taman A, Ribeiro P (2011) Glutamate-mediated signalling in *Schistosoma mansoni*: A novel glutamate receptor is expressed in neurons and the female reproductive tract. Mol Biochem Parasitol 176: 42–50. 21163308 10.1016/j.molbiopara.2010.12.001

[44] Mendonca-Silva DL, Pessoa RF, Noel F (2002) Evidence for the presence of glutamatergic receptors in adult *Schistosoma mansoni* . Biochem Pharmacol 64: 1337–1344. 12392816 10.1016/s0006-2952(02)01358-8

[45] Horman S, Morel N, Vertommen D, Hussain N, Neumann D, et al (2008) AMP-activated protein kinase phosphorylates and desensitizes smooth muscle myosin light chain kinase. J Biol Chem 283: 18505–18512. 18426792 10.1074/jbc.M802053200

[46] Mair GR, Maule AG, Day TA, Halton DW (2000) A confocal microscopical study of the musculature of adult *Schistosoma mansoni* . Parasitology 121: 163–170. 11085236 10.1017/s0031182099006174

[47] Swierczewski BE, Davies SJ (2010) Developmental regulation of protein kinase A expression and activity in *Schistosoma mansoni* . Int J Parasitol 40: 929–935. 20097200 10.1016/j.ijpara.2010.01.001 PMC2875359

[48] Ehlers MD (2000) Reinsertion or degradation of AMPA receptors determined by activity-dependent endocytic sorting. Neuron 28: 511–525. 11144360 10.1016/s0896-6273(00)00129-x

[49] Hammond RS, Lin L, Sidorov MS, Wikenheiser AM, Hoffman DA (2008) Protein kinase A mediates activity-dependent Kv4.2 channel trafficking. J Neurosci 28: 7513–7519. 18650329 10.1523/JNEUROSCI.1951-08.2008 PMC2665045

[50] Shaywitz AJ, Greenberg ME (1999) CREB: a stimulus-induced transcription factor activated by a diverse array of extracellular signals. Annu Rev Biochem 68: 821–861. 10872467 10.1146/annurev.biochem.68.1.821

[51] Matsuyama H, Takahashi H, Watanabe K, Fujimaki Y, Aoki Y (2004) The involvement of cyclic adenosine monophosphate in the control of schistosome miracidium cilia. J Parasitol 90: 8–14. 15040661 10.1645/GE-52R1

[52] Ressurreicao M, Rollinson D, Emery AM, Walker AJ (2011) A role for p38 MAPK in the regulation of ciliary motion in a eukaryote. BMC Cell Biol 12: e6. 10.1186/1471-2121-12-6 PMC3040701 21269498

[53] Taft AS, Norante FA, Yoshino TP (2010) The identification of inhibitors of *Schistosoma mansoni* miracidial transformation by incorporating a medium-throughput small-molecule screen. Exp Parasitol 125: 84–94. 20060828 10.1016/j.exppara.2009.12.021 PMC2859107

[54] Ludtmann MHR, Rollinson D, Emery AM, Walker AJ (2009) Protein kinase C signalling during miracidium to mother sporocyst development in the helminth parasite, *Schistosoma mansoni* . Int J Parasitol 39: 1223–1233. 19394337 10.1016/j.ijpara.2009.04.002

[55] Ressurreicao M, Rollinson D, Emery AM, Walker AJ (2011) A role for p38 mitogen-activated protein kinase in early post-embryonic development of *Schistosoma mansoni* . Mol Biochem Parasitol 180: 51–55. 21787807 10.1016/j.molbiopara.2011.07.002

